# Recent Advances in Biomass-Derived Activated Carbon and Biochar for Tetracycline Removal from Water: A Comprehensive Review

**DOI:** 10.1021/acsomega.6c00799

**Published:** 2026-06-03

**Authors:** Hari Om Singh, Ramesh Vinayagam, Raja Selvaraj

**Affiliations:** Manipal Institute of Technology, 76793 Manipal Academy of Higher Education, Manipal 576104, India

## Abstract

Here, we review the recent advancements in the use of sustainable carbon materials, namely, activated carbon (AC) and biochar (BC), for the adsorption of tetracycline (TC) from aqueous media. The environmental persistence of TC and its role in promoting antimicrobial resistance have necessitated the development of efficient, scalable, and sustainable treatment solutions. AC and BC, synthesized from agricultural and industrial biomass waste, offer high specific surface areas, tunable porosity, and surface functionalities that make them highly effective for TC removal. This review presents a comprehensive evaluation of biomass selection, synthesis routes, activation strategies, and surface modifications that influence the structural and chemical characteristics of these adsorbents. Key adsorption mechanisms such as π–π electron donor–acceptor interactions, hydrogen bonding, electrostatic interactions, surface complexation, and pore filling are critically discussed in relation to environmental parameters, including pH, ionic strength, and contaminant speciation. In addition, adsorption kinetics and isotherm models are analyzed to elucidate the underlying transport and binding processes. Finally, we identify current challenges and future opportunities in optimizing AC and BC for real-world applications in wastewater treatment systems. This review aims to serve as a scientific foundation for the rational design of next-generation carbon-based materials for effective antibiotic removal and environmental protection.

## Graphical abstract

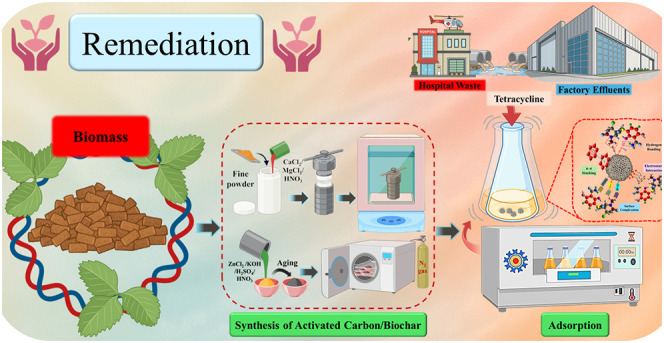

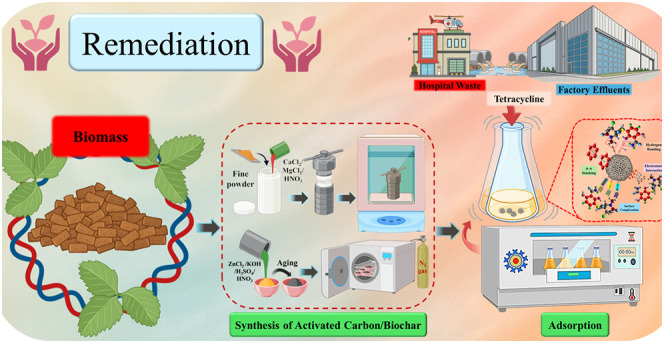

## 1 Introduction

Freshwater scarcity and water pollution are escalating global challenges driven by climate change, population growth, industrialization, and intensive chemical usage in healthcare, which threaten sustainable development and food security.^1^ Antibiotics have changed modern medicine by making many serious infections treatable. Among them, tetracycline (TC) is one of the most widely used because it is broad-spectrum, inexpensive, and effective.^2^ It continues to be produced in large quantities for both human and veterinary use. However, this heavy use has also increased the amount of TC that enters the environment. In many regions, conventional wastewater treatment plants are not designed to completely remove pharmaceuticals; therefore, effluents containing TC can reach natural water bodies. These discharges may contain unmetabolized TC, transformation products formed during use or treatment, and other pharmaceutical residues. Over time, such substances can persist and accumulate in water and soil, posing potential risks to ecosystems and public health, including the development and spread of antimicrobial resistance.^3^

Because a considerable fraction of TC is excreted in its active form, it can enter municipal and hospital wastewater. In anaerobic digestion systems, TC can undergo adsorption onto sludge as well as biodegradation by anaerobic microorganisms, which together govern its fate during wastewater treatment.^4^ In livestock systems, manure from treated animals is another important source; when this manure is stored or applied to fields, TC residues can runoff into surface waters or percolate into groundwater (Figure 1). Even though environmental concentrations are usually low, these residues can still disturb microbial communities and promote the selection and spread of antibiotic-resistance genes, which is a recognized public health concern.^2^ In natural waters and soils, TC binds strongly to natural organic matter and mineral surfaces, which influences its mobility, persistence, and bioavailability; uptake by organisms and possible transfer through the food chain raise further long-term ecological concerns.^5^ The relatively stable structure of TC and the presence of multiple functional groups also make it difficult to remove by using conventional treatment processes. Although awareness of pharmaceutical pollution is increasing, regulations remain limited: organizations such as the World Health Organization (WHO) and the Environmental Protection Agency (EPA) provide broad water-quality guidelines, but TC-specific discharge standards and routine monitoring requirements are often lacking.

**Figure 1:**
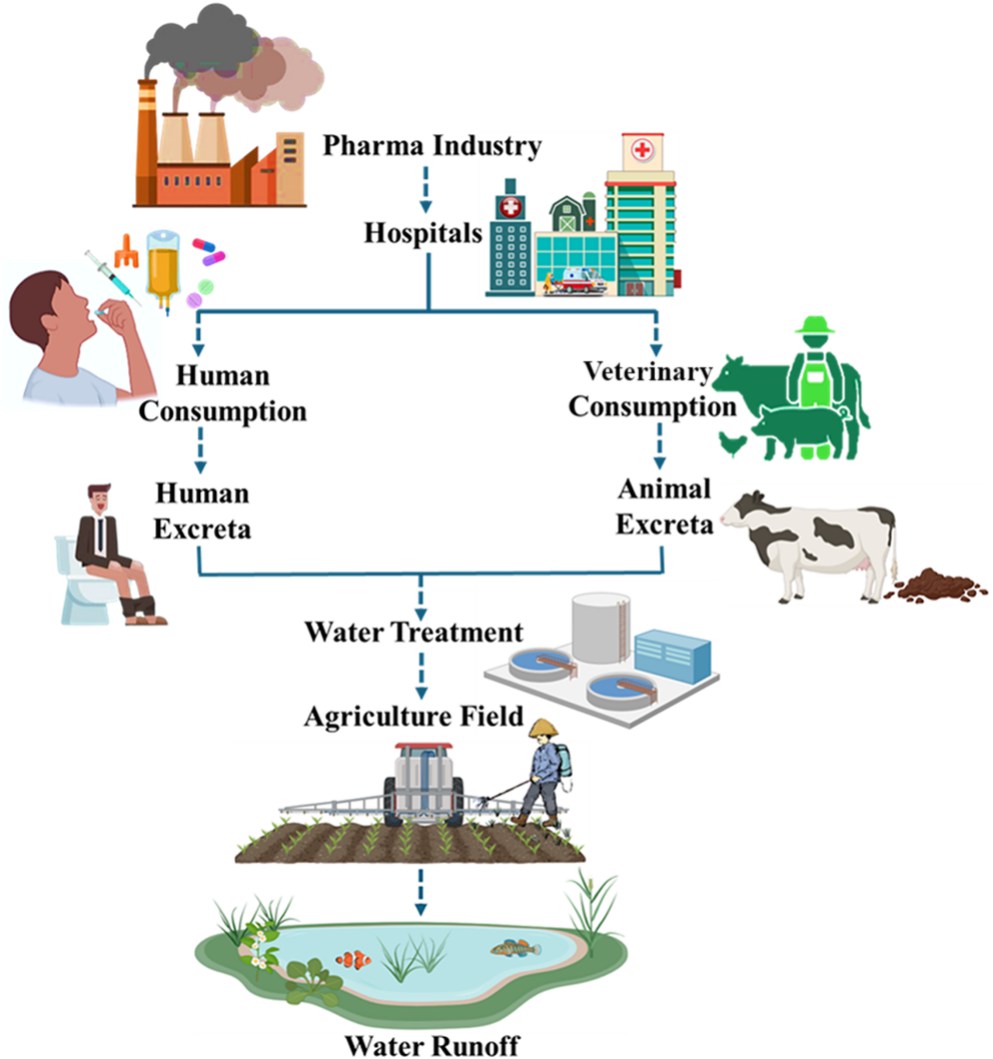
Pathways of TC contamination in the environment from anthropogenic and agricultural sources to water systems (Created in BioRender).

Several conventional methods are used to remove antibiotics from wastewater, such as electrochemical oxidation, filtration processes, catalytic degradation, membrane separation, ion exchange, and adsorption. Among these, adsorption is often preferred because the operation is simple, the energy demand is relatively low, and high removal efficiencies can be achieved without generating large amounts of secondary waste. For TC, a variety of adsorbents have been studied, including activated carbon (AC), biochar (BC), carbon nanotubes (CNTs), metal–organic frameworks (MOFs), zeolites, and graphene oxide. CNTs, MOFs, and graphene-based materials can provide very high specific surface areas and adjustable surface chemistry, but their large-scale use is limited by costly precursors, multistep synthesis routes, and concerns about environmental safety.^6^ Zeolites can remove contaminants by ion exchange and adsorption, but they usually show limited affinity for neutral or weakly polar organic molecules such as TC unless their surface is modified.^7^ In practical applications, AC and BC are still widely used because they are comparatively inexpensive, can be produced from abundant feedstocks, and have a good record in removing different pollutants. AC generally has a more developed pore structure and higher surface area, whereas BC is increasingly considered as a more sustainable option due to its low-cost and resource-efficient production.

Despite extensive work on TC adsorption, many reviews either discuss antibiotic removal at a generalized level or restrict their scope to individual adsorbent types, which reduces their relevance for real-world process design and optimization. In particular, there is still no clear side-by-side comparison of AC and BC for TC removal. Both materials have shown good potential, but current work rarely brings together information on their preparation routes, surface properties, pore structures, and functional groups in relation to adsorption performance.

This review aims to fill that gap by summarizing recent advances in the use of AC and BC for TC removal from water. It covers different synthesis and activation methods, including physical and chemical activation and low-temperature pyrolysis, and discusses key physicochemical features such as specific surface area, pore volume, and surface chemistry. Adsorption behavior is interpreted using common kinetic and isotherm models, with attention to mechanisms such as hydrogen bonding, π–π interactions, and electrostatic attraction. The effects of operating conditions, including pH, initial TC concentration, contact time, and temperature, are also considered. By linking adsorption performance with practical aspects of material preparation and use, this review offers a consolidated basis for developing efficient and sustainable AC- and BC-based adsorbents for TC removal in water treatment. This review focuses on TC as a representative compound of the TC-class antibiotics; extension to other derivatives (e.g., doxycycline) is beyond the present scope and is proposed as future work.

## 2 Methodology

A structured review was undertaken to compile evidence of biomass-derived AC and BC for TC removal from aqueous systems. The review considered biomass sources, synthesis/activation approaches, characterization outcomes, and the reported adsorption performance. The literature was retrieved from Scopus and Google Scholar by searching the title, abstract, and keyword fields with the Boolean query: (“activated carbon”) AND (“biochar”) AND (“tetracycline” AND “adsorption”) AND (“biomass”). The query was chosen to capture studies explicitly linking biomass-based AC/BC preparation to TC adsorption applications.

## 3 Persistence, Resistance, and Regulatory Implications of TC in Aquatic Systems

Since 2002, antibiotic use worldwide has expanded dramatically. Earlier reports described global antibiotic consumption in terms of mass, typically **∼**100,000–200,000 tonnes per year. By 2020, usage was increasingly quantified using the public-health metric defined daily doses (DDDs), with global consumption estimated at ∼4.5 trillion DDDs.^8^ Use of TC has also been substantial: the European Union documented around 2294 tons for medical purposes in 1997, and U.S. veterinary applications exceeded 3200 tons by 2001.^9^ After therapeutic use, a substantial fraction of TC is excreted unmetabolized. Because conventional wastewater treatment plants (WWTPs) are not specifically designed to fully remove such micropollutants, a portion can pass through treatment processes and be discharged into receiving waters. Its high-water solubility and low volatility favor persistence in aquatic systems, increasing the likelihood of prolonged exposure.

Continued inputs of TC and related compounds can encourage the emergence and spread of antibiotic-resistance genes (ARGs) through mutation and horizontal gene transfer in microbial communities. TC-resistance genes such as tet­(O) and tet­(S) have been detected in human gut microbiota and in various environmental samples, indicating potential public-health implications. In Europe, resistance levels as high as 66.9% in *Escherichia coli* and 44.9% in Klebsiella spp. have been reported.^10^ Studies from North America and Asia similarly describe the presence of genes including tet­(M), tet­(Q), and tet­(W) in wastewater, rivers, and waters affected by aquaculture, and *E. coli* from U.S. dairy manure has shown almost universal resistance.^11^ The global occurrence of TC in water bodies is supported by measured concentrations at different sites. Reported levels include 280–540 ng/L in Iranian municipal wastewater,^12^ 5.3 ng/L in WWTP effluent in North America,^13^ and 21 ng/L in the Soeste River in Germany.^14^ Such differences reflect regional usage patterns and the limits of current wastewater treatment systems. Residual TC concentrations in various water sources worldwide are summarized in Table 1.

**Table 1:** Residual Concentrations of TC in Different Water Sources

S.No	water source	reported TC concentration (ng/L)	country	refs.
1	Municipal wastewater	280 to 540	Iran	^12^
2	Wastewater treatment systems	5.3	North America	^13^
3	Soeste river	21	Germany	^14^
4	Hanoi Lake	101	Hanoi, Vietnam	^15^
5	Drinking water source	11.16	China	^16^
6	Hospital effluent	1385	Norway	^17^
7	Aquaculture water	180	Thailand	^18^
8	Hospital wastewater	42.2	Portugal	^19^
9	Urban water	100	Australia	^20^

The regulation of TC in water has therefore become an important environmental and public-health issue. Its persistence, widespread use, and ability to promote antibiotic resistance are central concerns. In many countries, however, specific regulatory limits for TC in drinking water and wastewater are still lacking. Where guidance exists, it generally aims to keep TC at trace levels, often in the ng/L to low μg/L range, to reduce long-term exposure and ecological pressure that favors resistant bacteria. As new toxicological and environmental data become available, regulatory agencies are moving toward stricter standards and more consistent monitoring of TC in different water bodies.

For example, Antos et al. reported that TC levels in European surface waters are generally in the 0–20 ng/L range.^21^ Zhou et al. examined TC at environmentally relevant concentrations (3.9–250 ng/mL) and showed that exposure within this window can increase the conjugative transfer of antibiotic-resistance genes (ARGs), potentially intensifying antimicrobial-resistance risks.^22^ At higher concentrations, Taşkan found that 0.25–30 mg/L TC can suppress sensitive algal populations, with implications for aquatic ecosystem function.^23^ Taken together, these studies indicate that TC may pose ecological and health-related concerns across a wide concentration range, including at trace levels. This evidence strengthens the case for treatment approaches that can reliably remove TC at low concentrations, particularly as monitoring expectations and discharge requirements evolve. Among the available options, AC and BC have received sustained interest because their adsorption behavior can be tuned through surface area, pore structure, and surface functional groups. In this review, we focus on biomass-derived AC and BC as practical sorbents for TC control in water systems. The next sections summarize their preparation routes and key properties, and then discuss how these factors translate into adsorption performance in aqueous media.

## 4 Biomass Feedstocks and Activation Methods

### 4.1 AC Synthesis

The performance of AC is closely linked to the nature of the biomass feedstock, since the precursor composition largely determines how the carbon framework and surface groups develop during activation. Many low-cost resources, such as agricultural residues, industrial byproducts, and food-processing wastes, have been converted into AC with good adsorption properties. In addition, ACs that are hybridized or surface-modified often show improved uptake because new functional groups and reactive sites can be introduced, creating a combined (synergistic) effect. In general, AC can be prepared from a wide range of carbon-rich materials, including coal, wood, nutshells, peat, and other plant-based residues, and its pore structure and surface chemistry can be tuned by the activation route. Prior to activation, the raw precursor shows typical lignocellulosic characteristics with abundant oxygen-containing groups.^24^ In chemical activation, the precursor is impregnated with reagents such as H_3_PO_4_, ZnCl_2_, KOH, H_2_SO_4_, or HNO_3_, followed by carbonization at 400–900 °C, which helps control surface functionalities and promotes micropore development.^25^

After carbonization, oxygen-containing surface groups such as carboxyl, hydroxyl, carbonyl, and C–O functionalities can be detected;^26^ moreover, chemical activation can generate distinct surface moieties, including −OH, −C=O, −P=O, and −P–O–C functionalities under H_3_PO_4_ activation,^27^ while KOH activation is often evidenced by the appearance of peaks associated with more oxygen-containing functional groups.^28^ The activating chemical plays a major role in shaping the final pore architecture and surface chemistry. In physical activation, the material is first carbonized under an inert atmosphere and then activated by gasification using steam or CO_2_ at 800–1100 °C, generating porosity through controlled burnoff; the resulting pore network depends on the activating gas and operating conditions.^29^

Among various studies, Yang et al. prepared AC from corn straw using a modified H_3_PO_4_ activation approach. Briefly, 10 g of corn straw was soaked in 120 mL of H_3_PO_4_, mixed for 30 min, sonicated for 1 h, and then dried at 105 °C overnight. The treated and untreated materials were subsequently pyrolyzed at 300 °C for 2 h under a nitrogen atmosphere, using a heating rate of 5 °C/min. After carbonization, the product was thoroughly washed, dried at 65 °C, and processed by grinding and sieving (60 mesh). The final AC showed a specific surface area of 463.89  m^2^/g and a pore volume of 0.387 cm^3^/g, confirming the formation of a more porous structure.^30^

In contrast, Wang et al. produced AC from semicoke using KOH as the activating agent. The semicoke was mixed with KOH at mass ratios from 1:0 to 1:3 and then activated under a nitrogen atmosphere at 773–1173 K. The resulting AC reached a specific surface area of 1365.79 m^2^/g and a pore volume of 0.38 cm^3^/g, reflecting substantial pore development. The method is relatively economical and can deliver strong adsorption performance; however, it requires high activation temperatures, and the adsorbent may lose efficiency after multiple regeneration cycles.^31^

In a similar approach, Islam et al. prepared a KOH-activated carbon (KOH–AC) from palm leaf biomass for TC adsorption. The raw biomass was first washed and milled, then sieved to obtain particles of about 125 μm. For activation, the material was soaked in 1 M KOH and subjected to ultrasonic treatment for 4 h to promote impregnation. The KOH-treated precursor was then carbonized at 800 °C under a nitrogen atmosphere (16 mL/min), using a heating rate of 10 °C/min. After carbonization, the solid was washed stepwise with 20% ethanol, 1 M HCl, and deionized water, and finally dried at 80 °C. The resulting AC showed a specific surface area of 540 m^2^/g and a pore volume of 0.261 cm^3^/g, suggesting that KOH activation produced a well-developed porous structure.^32^ The general AC preparation routes discussed above are schematically presented in Figure 2, and additional biomass-based AC studies for TC removal are compiled in Table 4.

**Figure 2:**
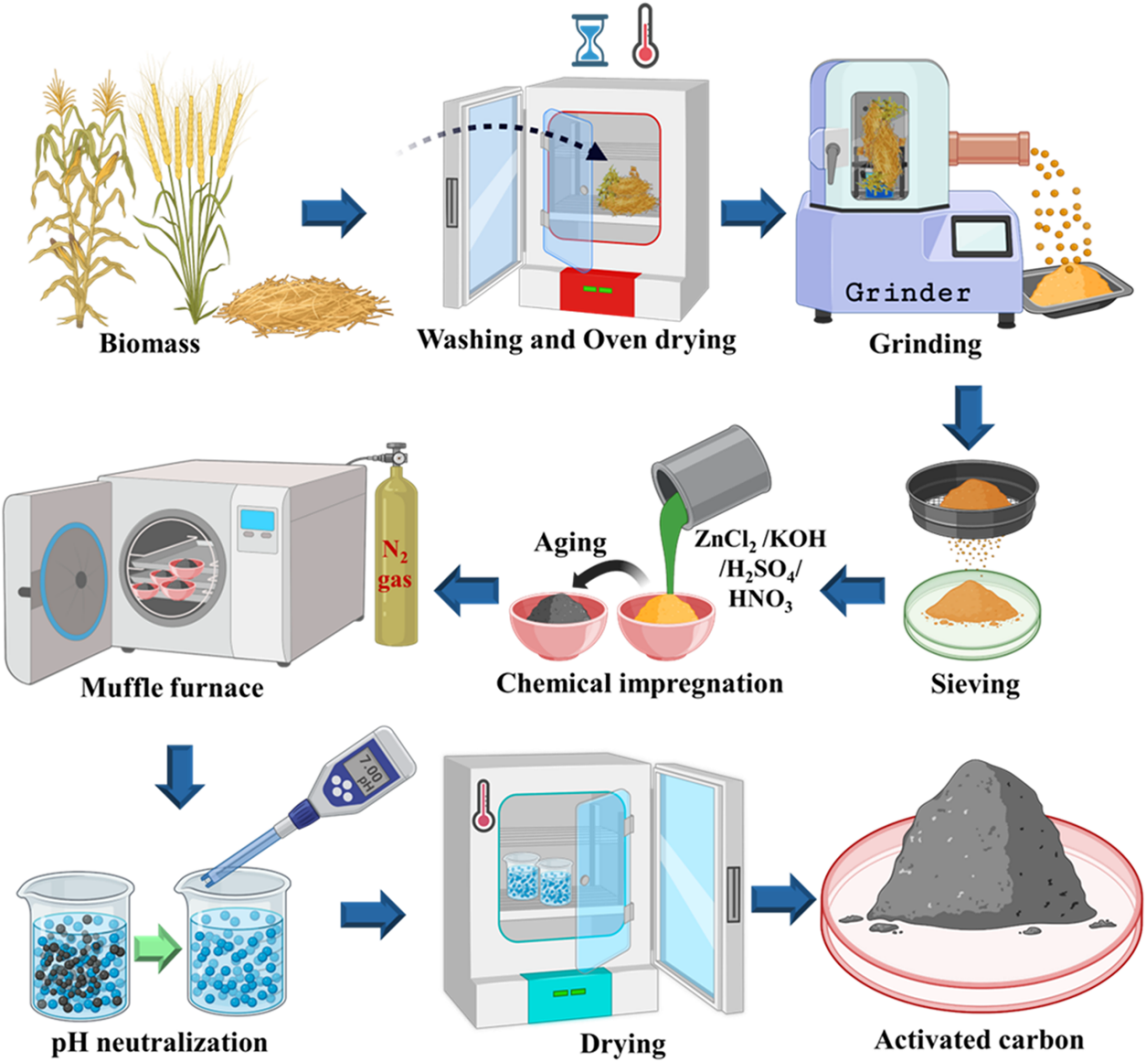
Biomass precursors and chemical activation for AC production (created in BioRender).

### 4.2 BC Synthesis

BC is a carbon-rich material commonly produced by pyrolysis of biomass under oxygen-limited conditions, typically at 300–700 °C. Interest in BC has increased because it can support sustainable agriculture, contribute to carbon storage, and serve as a low-cost material for environmental cleanup. During pyrolysis, the main biomass components—cellulose, hemicellulose, and lignin—break down through a series of overlapping reactions (e.g., dehydration and depolymerization followed by cracking, aromatization, and condensation). These steps gradually convert the feedstock into a more aromatic, porous, and chemically stable carbon matrix.^33^ Before pyrolysis, biomass feedstocks possess inherent oxygen-containing functional groups derived from their main lignocellulosic constituents. Specifically, cellulose is mainly enriched with hydroxyl (−OH) and C–O groups, hemicellulose contains carbonyl (C=O) functionalities, and lignin contains an abundance of methoxyl (−O–CH_3_), ether (C–O–C), and aromatic C=C groups.^34^ With increasing pyrolysis temperature, the H/C and O/C atomic ratios decrease.^35^ The properties of BC depend strongly on operating conditions and the type of biomass used. Parameters such as temperature, heating rate, residence time, and feedstock composition control pore development and surface chemistry, which in turn affect specific surface area, pore structure, and the abundance of surface functional groups. In particular, major structural changes in the 200–600 °C range are often associated with the onset of char formation and the development of a more carbon-rich framework. Besides conventional pyrolysis, other thermochemical routes, such as gasification, hydrothermal carbonization, torrefaction, and flash carbonization, are also used to suit different feedstocks and moisture levels.^33,36^ The hydrothermal approach is schematically shown in Figure 3, and recent reports on BC preparation conditions and resulting properties are compiled in Table 4.

**Figure 3:**
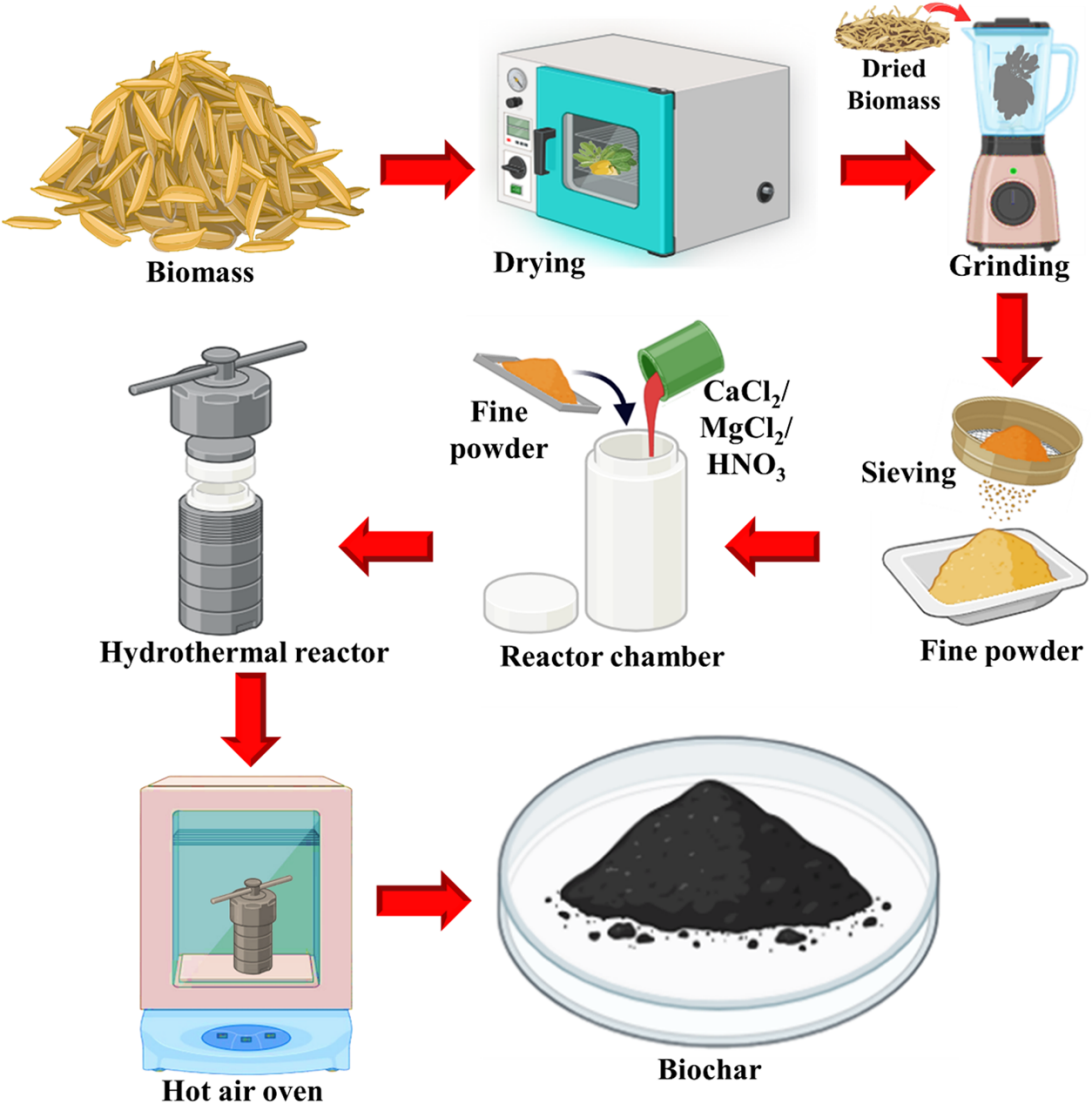
Biomass feedstocks and hydrothermal processing method for BC production (Created in BioRender).

The properties of BC depend strongly on how it is prepared, and this is clear from reported synthesis studies. For instance, Xiang et al. produced wheat-stalk BC and then improved it through physical processing. The feedstock was washed and dried, cut into ∼1 cm pieces, and pyrolyzed at 300, 450, and 600 °C for 4 h under nitrogen. The char was washed, dried at 105 °C, and sieved. It was then ball-milled at 300 rpm for 12 h, using a biochar-to-ball mass ratio of 1:100. Among the tested samples, the best material reached a specific surface area of 257.50 m^2^/g and a pore volume of 0.1966 cm^3^/g, showing that higher pyrolysis temperature combined with ball milling can substantially improve surface development.^37^ Likewise, Shao et al. prepared BC from cow dung using both one-step and two-step pyrolysis routes. In the single-step approach, the material was heated to 900 °C under nitrogen to obtain a BC with improved structural features. The two-step method used sequential heating with controlled holding periods, either 700 °C followed by 900 °C or the reverse order (900 → 700 °C). The BCs produced by these routes showed improved performance for TC adsorption.^38^

For example, Miao et al. produced phosphoric-acid-activated BC from peanut shells (PS) by varying the PS/H_3_PO_4_ mass ratio from 1:1 to 1:4. The shells were washed, dried, and sieved before impregnation with H_3_PO_4_. After ultrasonication, the material was dried at 150 °C and then pyrolyzed at 800 °C for 2 h under nitrogen. Under the optimized condition, the resulting BC reached a specific surface area of 1753 m^2^/g with a pore volume of 1.2972 cm^3^/g, demonstrating the strong pore-forming effect of H_3_PO_4_ activation.^39^ In a similar manner, Qin et al. prepared KOH-activated BC using rape straw. The precursor was ground and passed through a 200-mesh sieve and first pyrolyzed at 700 °C for 2 h under argon. The obtained BC was then blended with KOH at mass ratios of 1:1, 1.5:1, and 2:1, followed by sonication, drying at 120 °C, and a second heat treatment at 750 °C for 2 h. After activation, the products were washed with 1 mol/L HNO_3_ and deionized water and dried at 80 °C. The best-performing sample showed a specific surface area of 1531 m^2^/g and a pore volume of 1.0276 cm^3^/g, confirming effective porosity development through KOH activation.^40^

## 5 Factors Affecting TC Adsorption

Adsorbent performance for TC removal depends on both the material itself and the water matrix in which it is applied. In most studies, the main operating variables are temperature, pH, adsorbent dose, and background constituents such as salts, natural organic matter, or other dissolved compounds. Higher temperature often speeds up diffusion and transport of TC toward the surface, which can increase uptake when mass transfer is a limiting step. pH is equally influential because it controls the charge on the adsorbent surface and the dominant form of TC in solution, thereby shifting electrostatic effects and the strength of surface binding. In real waters, coexisting organics/inorganics, and in some cases microbial activity, can reduce adsorption by competing for sites, blocking pores, or changing local surface conditions. For consistent TC removal, these parameters must be evaluated together rather than in isolation.

### 5.1 Influence of Specific Surface Area

A larger SSA generally offers more available sites for TC adsorption and improves the contact between the adsorbent and the contaminant. In AC, the pore network usually contains both micropores and mesopores, depending on the raw material and the activation procedure. Micropores mainly account for the high SSA and adsorption capacity, whereas mesopores help TC molecules diffuse into the structure and reach the inner surface more easily.^41^ BC, on the other hand, often has an SSA lower than that of AC but still shows good adsorption performance because of its wider pore size distribution and the presence of various surface functional groups. In BC, the SSA is strongly influenced by factors such as pyrolysis temperature and feedstock type, which together control pore development and structural changes during carbonization.^42^

In the context of AC, Demiral et al. synthesized H_3_PO_4_–AC from peach fruit stones, achieving a maximum SSA of 1399 m^2^/g at 400 °C with a 3:1 acid-to-precursor ratio. Increasing the activation temperature to 600 °C reduced the SSA due to pore widening, while subsequent HNO_3_ treatment enhanced mesoporosity by collapsing micropores.^43^ Similarly, Jawad et al. reported a substantial increase in SSA from 0.47 to 681.4  m^2^/g after chemically activating Mangosteen peels.^44^

For BC-based adsorbents, Liu et al. reported that increasing the pyrolysis temperature increased the SSA, reaching 214.97 m^2^/g at 600 °C, and that an additional acid treatment further raised it to 319.80 m^2^/g.^45^ Saghir et al. also observed a strong rise in the SSA of pistachio shell-derived BC, from 15.12 m^2^/g at 300 °C to 467.8 m^2^/g at 900 °C, due to better pore development at higher temperatures.^46^ These results show that synthesis conditions have a major effect on the SSA of BC, which in turn improves adsorption efficiency. In general, an increase in SSA in carbonaceous adsorbents, whether AC or BC, provides more active sites for TC molecules and supports more effective removal from water. To facilitate comparison and highlight the SSA–performance relationship, Table 2 compiles representative AC and BC adsorbents with their reported textural properties and TC removal metrics. As summarized in Table 2, KOH-activated BCs exhibit a broad SSA range (132.82–3463.87 m^2^/g) with corresponding TC uptake from 86.95 to 2242 mg/g, indicating that stronger activation/pyrolysis conditions markedly enhance accessible porosity and adsorption. ACs also show wide variability (10.19–3037 m^2^/g; *q*_e_ = 132.94–1235 mg/g), and the data set emphasizes that TC uptake is governed not only by SSA but also by pore architecture and accessibility. For instance, even low SSA can still deliver high *q*_e_ when pore volume is large (e.g., oily sludge: 2.07 cm^3^/g^47^) and when surface chemistry provides strong specific interactions with TC.

**Table 2:** Effect of SSA and PV on *q*_e_ during TC Adsorption by Selected AC and BC Adsorbents

feedstock	synthesis method	activating agent	preparation temperature and time	SSA (m^2^/g)	pore volume (cm^3^/g)	*q*_e_ (mg/g)	ref.
**Biochar**							
Corn straw	Chemical	KOH	500 °C, 2 h	132.82	0.1589	86.95	^48^
Peanut shell	Chemical	KOH	600 °C, 2 h	1271.97	0.653	356.19	^49^
Rape straw	Chemical	KOH	700 °C, 2 h	1531	1.0276	325.07	^40^
Walnut shell	Chemical	KOH	900 °C, 1 h	1713.87	0.3	607.00 ± 31.87	^50^
Furfural residue	Chemical	KOH	800 °C, 1 h	3463.87	2.388	2242 ± 7.8	^51^
**Activated Carbon**							
Palm leaves	Chemical	KOH	800 °C	540	0.261	132.94	^32^
Oily sludge	Chemical	KOH	400 °C, 30 min	10.1938	2.07	205.1	^47^
Semicoke	Chemical	KOH	800 °C, 3 h	1365.79	0.38	302.99	^31^
*Forsythia Fructus* residue	Chemical	KOH	800 °C, 1 h	1625.62	0.78	754.66	^52^
Natural rock asphalt	Chemical	KOH	800 °C, 2 h	3037	2.122	1235	^53^

### 5.2 Impact of pH

The adsorption of TC on carbon-based adsorbents is strongly affected by the solution pH because it changes both the speciation of TC and the surface charge of the adsorbent. TC behaves as an amphoteric compound with three dissociation constants (p*K*_a1_ ≈ 3.4, p*K*_a2_ ≈ 7.7, and p*K*_a3_ ≈ 9.7), and its ionic form therefore varies with solution pH. At pH < 3.4, TC mainly exists as the cationic species (H_4_TC^+^); between pH 3.4 and 7.7, it occurs primarily as H_3_TC; between pH 7.7 and 9.7, it appears as H_2_TC^–^; and at pH > 9.7, it is mainly present as HTC^2–^.^54^ When the solution pH is below the pH_pzc_, the adsorbent surface is positively charged; at pH values above the pH_pzc_, the surface becomes negatively charged.

Above pH_pzc_, the pH response is governed by surface functional groups. Native AC/BC typically contains −OH/phenolic, −COOH/–COO^–^, >C=O, and C–O groups, whose protonation–deprotonation determines surface charge and hydrogen-bonding capacity. During carbonization, these oxygen-containing groups transform significantly,^55^ while activation introduces new functionalities such as C–O–P moieties from H_3_PO_4_ activation^56^ and redistributed oxygenated sites from KOH activation followed by acid washing. Further modifications, including oxidation and N-functionalization, introduce additional −COOH/–OH and N-containing sites.^57^ This directly affects electrostatic interactions with the different TC species. At low pH, both TC and the adsorbent surface are positively charged, which leads to electrostatic repulsion and lower adsorption. Around neutral pH, TC is mainly in its zwitterionic form and the surface charge of the adsorbent is weaker or mixed, so electrostatic repulsion is reduced and adsorption often increases due to hydrogen bonding, π–π interactions, and other nonelectrostatic forces.^32^ The Henderson–Hasselbalch equation can be used to estimate the distribution of TC species at different pH values and, together with the pH_pzc_ of the adsorbent, to relate speciation to adsorption trends. At alkaline pH, TC is mainly anionic, and the negatively charged surface tends to repel it, which lowers electrostatic adsorption; however, surface functional groups and secondary interactions can still support some uptake.^58^ In addition, metal ions such as Cu^2+^ can modify TC adsorption by forming TC–metal complexes under different pH conditions, which may either promote or reduce adsorption depending on the chemistry of the system.^59^

The pH-dependent nature of TC adsorption has been confirmed by various studies investigating different adsorbent systems. For instance, Cai et al. found that ZnCl_2_-modified material (ZBAC) showed a gradual decline in TC removal efficiency as pH increased, which was mainly attributed to the deactivation of functional groups and the emergence of electrostatic repulsion once the solution pH surpassed the pH_pzc_ of 8.3.^60^ Similarly, Ninh et al. found that Soybean curd residue-derived carbon (pH_pzc_ = 7.01) maintained stable TC removal across a wide pH range, suggesting dominance of non-electrostatic interactions.^61^

Xiang et al. studied wheat stalk BC and found that TC adsorption was highest near neutral pH, where zwitterionic TC interacts more easily with the surface because electrostatic repulsion is low. At higher pH, adsorption decreased due to repulsion between anionic TC and the negatively charged BC surface.^37^ Shao et al. worked with cow dung-derived BCs (pH_pzc_ 8.25–8.64) and observed maximum TC removal (∼88–93%) at pH 3.0. In this case, the expected electrostatic repulsion was offset by strong π–π interactions and hydrogen bonding. Adsorption was almost unchanged from pH 4 to 9, but dropped sharply above pH 9.^38^ Li et al. showed that surface modification can change the pH response. For KHCO_3^–^_ modified tea waste BC (pH_pzc_ = 9.23), the adsorption capacity decreased with increasing pH. At pH 7, it still reached 101.44 mg/g, indicating that nonelectrostatic interactions make an important contribution even near or above the pH_pzc_.^62^

#### 5.2.1 Determination and Significance of pH_pzc_

The pH_pzc_ represents the pH at which the adsorbent surface has no net charge and plays a key role in determining electrostatic interactions during adsorption. The pH_pzc_ is commonly determined using the pH drift method, where a series of inert electrolyte solutions (e.g., 0.01 M NaCl) are adjusted to different initial pH values. A fixed amount of adsorbent is added to each solution, and after equilibrium, the final pH is measured. The pHpzc corresponds to the point where the initial and final pH values are equal.^63^ Another frequently reported technique is ζ-potential measurement, where the surface electrokinetic potential is measured as a function of pH.^64^ The pH_pzc_ is strongly influenced by the balance of acidic and basic surface functional groups such as −COOH, phenolic −OH, carbonyl (>C=O), and nitrogen-containing moieties introduced during activation or modification.^65^ Thermal treatment, chemical activation, and postmodification alter the abundance and strength of these functional groups, thereby shifting the pH_pzc_.

### 5.3 Effect of Dosage

Adsorbent dosage strongly affects how much TC can be removed from water, because it controls the availability of active adsorption sites. At low dosages, there are not enough sites, so TC removal is often incomplete. As the dosage increases, removal usually improves because more surface area and pores are available. However, after a certain point, adding more adsorbent can cause active site overlap and lower the adsorption capacity per unit mass.^66^ It is therefore important to find an optimum dosage that gives good TC removal without unnecessary material use. This optimization depends on the properties of the adsorbent, the solution conditions, and the initial TC concentration.

Several studies have reported the effect of adsorbent dosage on TC removal using AC and BC. Sayğılı and Güzel tested tomato waste-based AC by changing the dosage from 10 to 50 mg in 50 mL of a 100 mg/L TC solution at 298 K, natural pH, and 1 h contact time. As the dosage increased, the adsorption capacity decreased from 92.5 to 73.7 mg/g, which they related to the aggregation of particles and the overlap of active sites. Based on these results, 50 mg was chosen as the working dosage for further experiments.^67^ For BC-based materials, Zhang et al. studied pristine and ball-milled BC prepared from crayfish shells. When the BC dosage increased from 10 to 100 mg, the adsorption capacity dropped from 110.1 to 25.6 mg/g for pristine BC and from 122.6 to 35.2 mg/g for ball-milled BC, mainly because fewer TC molecules were available per unit mass at higher dosages. In contrast, the overall removal efficiency increased from 27.5 to 64.1% for pristine BC and from 30.7 to 88.0% for ball-milled BC. The higher removal in the ball-milled sample was ascribed to its larger SSA and better exposure and dispersion of active sites, which supported more effective TC adsorption.^68^

### 5.4 Effect of Initial TC Concentration

The initial concentration of TC in solution is an important factor that controls how carbon-based adsorbents perform. It directly affects the concentration gradient that drives the TC from the bulk solution to the adsorbent surface. At low TC concentrations, there are many free active sites compared with the number of TC molecules, so the removal efficiency is usually high. As the concentration increases, these sites gradually become occupied and the removal efficiency starts to fall. In contrast, the adsorption capacity often increases with increasing TC concentration because the stronger driving force for diffusion promotes uptake until a plateau is reached when the adsorbent is saturated.^69^ This behavior is seen not only for AC but also for BC, especially for materials with well-developed pores and abundant surface functional groups. At very high TC concentrations, competition for sites and limited access to inner pores can slow mass transfer and reduce overall efficiency.^70^ Understanding the role of the initial TC concentration is therefore important for optimizing adsorption systems, interpreting adsorption isotherms, and designing TC removal processes for water treatment.

Several studies have examined how the starting TC concentration affects the removal by different ACs. Nayak et al. investigated the effect of the initial tetracycline concentration on adsorption by activated carbon by varying the concentration from 100 to 1000 mg/L at a fixed adsorbent dose of 1.25 g/L. They reported that the adsorption percentage decreased as the initial antibiotic concentration increased.^71^ Ghorbanian et al. observed a similar trend for AC prepared from hard pistachio shells: as the initial TC concentration increased from 10 to 50 mg/L, the removal efficiency decreased from 68.6 to 30.2%.^72^

Similar trends have been reported for BC-based adsorbents. Naghipour et al. studied TC removal using 1 g of BC at pH 5.1 with initial concentrations from 50 to 500 mg/L. As the concentration increased, removal efficiency decreased, but the adsorption capacity rose from 62.5 to 75.6 mg/g, meaning that although the percentage removal fell, more TC was taken up per gram of BC.^73^ Zhao et al. examined TC adsorption on BCs prepared at different temperatures. When the initial TC concentration increased from 50 to 500 mg/L, the adsorption capacity of 450-B increased from 4.24 to 11.30 mg/g, and that of 650-K from 91.82 to 173.61 mg/g. At higher concentrations, the increase in capacity became smaller, showing gradual saturation of adsorption sites and a near-plateau in uptake.^74^

### 5.5 Effect of Contact Time

Contact time is the period during which TC molecules remain in contact with the adsorbent surface and has a direct effect on adsorption. In most cases, adsorption occurs in two steps: a fast initial stage, when many active sites are available, followed by a slower stage as these sites become filled. The drop-in rate at the later stage is usually linked to pore diffusion limits or surface saturation.^75^ The time required to reach adsorption equilibrium depends on the accessibility of active sites and the diffusion of TC molecules within the pore network of the adsorbent. Structural features such as pore architecture and surface properties can therefore influence adsorption/desorption behavior and the overall equilibration process.^76^

Liu et al. explored TC adsorption on porous biomass carbon prepared from garlic skin. They reported a very fast uptake within the first 5 min, followed by a slower stage, with equilibrium reached after 24 h. The adsorption capacity was 1588.9 mg/g at an initial TC concentration of 400 mg/L and increased to 1911.8 mg/g at 800 mg/L, showing the strong affinity of this material for TC.^77^ Ninh et al. investigated mesoporous AC from soybean curd residue and found that about 80% of TC was removed within the first 30 min; adsorption then continued beyond 180 min, indicating that, in the later stage, the process was mainly controlled by diffusion of TC into the mesopores.^61^ Saremi et al. examined vitamin B6-modified BC and observed a rapid rise in adsorption capacity to 54.9 mg/g within 10 min, after which the uptake curve flattened, indicating quick attainment of equilibrium.^78^ Together, these results show how contact time, pore structure, and surface modification govern both the rate and extent of TC adsorption.

### 5.6 Influence of Temperature

Temperature has a strong effect on TC adsorption onto carbon-based materials because it changes diffusion rates, surface interactions, and the properties of the adsorbent itself. In many cases, increasing the temperature raises the adsorption capacity, as higher thermal energy promotes molecular motion, speeds up intraparticle diffusion, and helps activate surface sites, which is consistent with an endothermic process.^79^ In other systems, adsorption behaves exothermically, especially when it is mainly physisorption or when strong adsorbate–adsorbent interactions release more energy than is needed to desolvate TC. Under those conditions, higher temperatures can lower adsorption efficiency. Thermodynamic parameters such as Gibbs free energy (Δ*G* ^0^), enthalpy (Δ*H* ^0^), and entropy (Δ*S* ^0^) are used to assess whether adsorption is spontaneous and to understand the heat effects involved. Carrying out experiments at different temperatures is therefore important for identifying the adsorption mechanism and selecting suitable operating conditions for TC removal.

Marzbali et al. observed a significant rise in the distribution coefficient (*K*_d_) from 5.34 to 34.69 as temperature increased from 308.15 to 338.15 K, due to the expansion of active sites. Thermodynamic analysis revealed an endothermic process (Δ*H*° = 54.02 kJ/mol) with increased disorder (Δ*S*° = 189.51 J/mol·K). Negative Δ*G*° values (−4.29 to −9.96 kJ/mol) indicated spontaneous adsorption that became more favorable at higher temperatures, likely driven by chemical interactions such as hydrogen bonding and protonation.^80^ Naghipour et al. found that TC removal efficiency increased from 10 to 50 °C. Thermodynamic data from Van’t Hoff plots showed an endothermic process with Δ*H*° = 38.74 kJ/mol and Δ*S*° = 137.09 J/(mol K). Δ*G*° values became increasingly negative (−0.78 to −6.28 kJ/mol) with rising temperature, confirming enhanced spontaneity.^73^

### 5.7 Effect of Coexisting Ions on Adsorption

The ions present in natural waters and wastewaters can strongly affect TC adsorption. Divalent cations such as Ca^2+^ and Mg^2+^ often reduce adsorption capacity by forming soluble complexes with TC or by occupying surface sites, whereas monovalent ions like Na^+^ and K^+^ usually interact more weakly and cause only limited interference.^81^ Common anions (NO_3_^–^, SO_4_^2–^, PO_4_^3–^) can also influence adsorption by changing the ionic strength of the solution and thus the electrostatic forces between TC and the adsorbent.^82^ Kim et al. studied the effect of coexisting ions on TC adsorption using coconut shell-based powdered AC and its thermally treated form at 800 °C. They reported that thermal treatment modified the surface functional groups and shifted the surface charge of the carbon, making it more positively charged under the experimental pH; this enhanced electrostatic attraction with the ionized TC species led to higher adsorption efficiency.^83^ BC-based adsorbents often show stronger sensitivity to ionic strength. Ma et al. examined furfural residue BCs produced at 300 and 600 °C and found that, as Na^+^ concentration increased from 0 to 0.1 mol/L, TC adsorption decreased from 5887 to 2415 mg/kg and from 9584 to 7565 mg/kg, respectively. Divalent cations (Ca^2+^ and Mg^2+^) at 0.02 mol/L suppressed adsorption even more than Na^+^, with Ca^2+^ causing about 48% greater reduction in TC uptake compared with Na^+^.^84^

## 6 Adsorption Models for TC Uptake

Understanding the kinetics and equilibrium behavior of TC adsorption on carbon-based adsorbents is important for designing practical water treatment systems. The main kinetic and isotherm models used for TC adsorption are summarized in Table 3. Kinetic models such as the pseudo-first-order (PFO) and pseudo-second-order (PSO) are commonly applied to describe how fast adsorption occurs and to infer the controlling mechanism. The PFO model is usually associated with physical adsorption driven by weak van der Waals forces, whereas the PSO model is more consistent with chemisorption involving electron transfer or sharing with surface groups such as −OH, −COOH, and C=O. The Elovich model is often used for chemisorption on heterogeneous surfaces with a wide range of activation energies, and the intraparticle diffusion (IPD) model helps to identify diffusion-controlled steps and boundary layer effects.^85^ In addition, the two-compartment first-order model describes adsorption as a two-stage process, with a fast initial step followed by a slower stage, providing a more detailed view of multistage adsorption behavior.^69^

**Table 3:** Various Kinetic, Isotherm, and Thermodynamic Models Applied in TC Adsorption

model	model equation	parameters and constants
**Kinetic Models**		
Pseudo-first-order (PFO)	$q_{t}=q_{e}\left(1-\exp\left({-K}_{1}t\right)\right)$	*q*_t_: adsorption capacity at time *t* (mg/g)
		*q*_e_: adsorption capacity at equilibrium (mg/g)
		*t*: contact time (min)
		*K*_t_: rate constant of the PFO (min^–1^)
Pseudo-second order (PSO)	$q_{t}=\frac{q_{e}^{2}K_{2}t}{{q_{e}K}_{2}t+1}$	*K*_2_: rate constant of the PSO
		(g.mg^–1^ min^–1^)
Intraparticle Diffusion	$q_{t}=Kt^{0.5}+C$	*K*: rate constant of IPD (mg g^–1^ min^0.5^)
		*C*, intercept constant (mg/g)
Elovich	$q_{t}=\frac{1}{\beta }\ln\left(\alpha \beta \right)+\frac{1}{\beta }\ln t$	β: desorption constant (g/mg)
		α: initial adsorption rate (mg/g·min)
**Isotherm Models**		
Langmuir	$q_{e}=\frac{q_{\max}bC_{e}}{{(1+\mathrm{bC}}_{e})}$	*q*_max_: maximum adsorption capacity (mg/g)
		*b*: Langmuir constant (L/mg)
Freundlich	$q_{e}=K_{F}C_{e}^{1/n}$	*K*_F_: Freundlich constant ((mg/g)/(mg/L)^1/*n*^)
		*C*_e_: equilibrium concentration (mg/L)
		*n*: Freundlich exponent (dimensionless)
Dubinin–Radushkevich	$q_{e}=q_{\max}\exp\left(-\beta \varepsilon^{2}\right)$	β: sorption energy constant (mol^2^/kJ^2^)
		ε: Polanyi potential (J/mol)
**Thermodynamic Models**		
Van’t Hoff Equation	$K_{T}=\exp\left[(\frac{\Delta S^{{}^{\circ}}}{R})-(\frac{\Delta H^{{}^{\circ}}}{R})\frac{1}{T}\right]$	*K*_T_: distribution factor (L/mg)
		Δ*S*°: standard entropy (J/mol K)
		Δ*H*°: standard enthalpy (kJ/mol)
		*R*: ideal gas constant (J/mol K)
		*T*: Absolute Temperature (K)

Adsorption equilibrium is usually described using different isotherm models. The Langmuir model assumes monolayer adsorption on a uniform surface, whereas the Freundlich model describes multilayer adsorption on heterogeneous surfaces. The Temkin model takes into account interactions between the adsorbate and adsorbent by assuming that the adsorption energy decreases linearly with coverage.^86^ The Dubinin–Radushkevich (D–R) model is used to distinguish between physical and chemical adsorption based on the mean adsorption energy and is also helpful for analyzing pore-filling processes. Using these models together helps to clarify the adsorption mechanism and to design AC- and BC-based systems for TC removal.^87^

Zhao et al. studied TC adsorption on granular AC using several kinetic models. Among them, the two-compartment first-order model gave the best fit to the data (*R* ^2^ = 0.9599). This model describes a two-step process with a fast initial uptake followed by a slower stage controlled by stronger chemisorption. In other words, TC is adsorbed quickly at first, and then a slower but more strongly bound fraction develops over time. The IPD model also indicated that adsorption occurs in more than one step, as the plots did not pass through the origin, showing the role of surface resistance and boundary layer effects. For the equilibrium data, the Temkin isotherm gave a good fit (*R* ^2^ = 0.8949), suggesting that interactions between TC and the AC surface, such as electrostatic attraction or ion exchange, play an important role in the adsorption process.^69^

Islam et al. studied TC adsorption on KOH-activated AC prepared from palm leaves. Kinetic data were best described by the PSO model, with a high correlation coefficient (*R* ^2^ = 0.970) and good agreement between experimental (*q*_e,exp_ = 94.00 mg/g) and calculated (*q*_e,cal_ = 96.75 mg/g) capacities, suggesting that the process is mainly chemisorption. For equilibrium, the adsorption data were well fitted by the Langmuir model (*R* ^2^ = 0.947), indicating monolayer coverage on a relatively uniform surface, with a maximum capacity (*q*_m_) of 132.94 mg/g at 25 °C. The Temkin model also showed a similar *R* ^2^ value (0.946), supporting the conclusion that the adsorption is endothermic.^32^ A comparative summary of ACs from different biomass sources is given in Table 4.

**Table 4:** Comparative Synthesis Methods and Adsorption Performance of Biomass-Derived AC for TC Removal, Including Isotherm and Kinetic Model Analysis

feedstock	synthesis method	activating Agent	SSA (m^2^/g)	removal (%)	*q*_max_ (mg/g)	isotherm model	kinetic model	thermodynamic nature	ref.
*Thysanolaena maxima*	Chemical	KOH	1065.011	97	21.317	Langmuir	Pseudo-second-order	Endothermic	^96^
**Commercial AC (Copper sulfate-impregnated)**	Chemical	Cu	533.5	—	34.12	Langmuir	—	Exothermic	^97^
**Palm leaves**	Chemical	KOH	540	88.18	132.94	Langmuir	Pseudo-second-order	Endothermic	^32^
**Corn Straw**	Chemical	H_3_PO_4_	463.89	98	227.3	Langmuir	Pseudo-second-order	Endothermic	^30^
**Waste plastics**	Chemical	KOH, NaCl	1006.31	100	242.46	Langmuir	Pseudo-second-order	Endothermic	^98^
**Semicoke**	Chemical	KOH	1365.79	—	315.12	Langmuir	Pseudo-second-order	—	^31^
**Sawdust**	Chemical	P_2_O_5_	1579	—	523.63	Langmuir	Pseudo-second-order	Endothermic	^99^
**Wheat Dust**	Chemical	NaOH	2821.58	—	1414.65	Freundlich	Pseudo-second-order	Endothermic	^100^
**Straw**	Chemical	K_2_CO_3_	1580	90	615	Langmuir	Pseudo-second-order	Endothermic	^101^

Extending the understanding of BC systems, Shi et al. studied TC adsorption on walnut shell-derived BC using kinetic, isotherm, and diffusion models. Among the kinetic models, the PSO model fitted the data better than the PFO model, indicating that chemisorption is the main rate-controlling step. The IPD and film diffusion models showed that adsorption proceeded in several stages. To analyze equilibrium behavior, the data were fitted to both Langmuir and Freundlich isotherms. The Langmuir model gave the better fit (*R* ^2^ = 0.95), suggesting monolayer adsorption on a relatively uniform surface with a finite number of identical sites.^50^

Lin et al. examined TC adsorption on BC derived from *Schizochytrium limacinum*. The kinetics followed the PSO model (*R* ^2^ = 0.99), again pointing to chemisorption. In this case, the Freundlich isotherm described the equilibrium data more accurately (*R* ^2^ = 0.96), while the Langmuir model estimated a high monolayer capacity of 1357.5 mg/g, indicating strong adsorption potential for TC removal.^88^ A broader comparison of BC adsorbents and their performance is given in Table 5.

**Table 5:** BC Adsorbents from Various Biomass Precursors with Key Synthesis Details and TC Adsorption Parameters^a^

feedstock	synthesis method	activating Agent	SSA (m^2^/g)	removal (%)	*q*_max_ (mg/g)	isotherm model	kinetic model	thermodynamic nature	ref.
**Wheat stalk**	Physical	N_2_	257.50	—	75.95	Langmuir	Pseudo-second-order	—	^37^
**Cow dung**	Physical	N_2_	238.93	98.96	158.9322	Langmuir	Pseudo-second-order	—	^38^
**Pinus taeda**	Chemical	NaOH	959.9	—	274.8	Freundlich	Elovich	Exothermic	^104^
**Tea waste**	Chemical	KHCO_3_	1981	—	293.46	Freundlich	Pseudo-second-order	—	^62^
**Alfalfa**	Chemical	NaOH	796.50	—	302.37	Langmuir	Pseudo-second-order	—	^105^
**Rape straw**	Chemical	KOH	1531	97	325.07	Freundlich	Pseudo-second-order	Endothermic	^40^
**Peanut shell**	Chemical	H_3_PO_4_	1753	—	570.73	Langmuir and Temkin	Elovich	—	^39^
**Walnut shell**	Chemical	KOH	1713.87	>95%	607.00	Langmuir	Pseudo-second-order	Endothermic	^50^
**Corn starch**	Physical and Chemical	ZnCl_2_	1645	100	1122.2	Freundlich	Pseudo-second-order	Endothermic	^106^
*Schizochytrium limacinum* **(microalgae)**	Chemical	KHCO_3_	2295.26	—	1357.5	Freundlich	Pseudo-second-order	—	^88^

a “Physical” = thermal/pyrolysis without chemical activators; “chemical” = activation/modification with chemical agents (e.g., KOH, ZnCl_2_, H_3_PO_4_, metal salts).

## 7 Adsorption Mechanism of TC

TC adsorption on carbon-based materials takes place through several physical and chemical interactions, such as π–π stacking, hydrogen bonding, electrostatic attraction or repulsion, hydrophobic effects, van der Waals forces, and pore filling (Figure 4). The dominance of these interactions depends on the surface chemistry, pore structure, and functional groups of the adsorbent, as well as solution conditions like pH and ionic strength.

**Figure 4:**
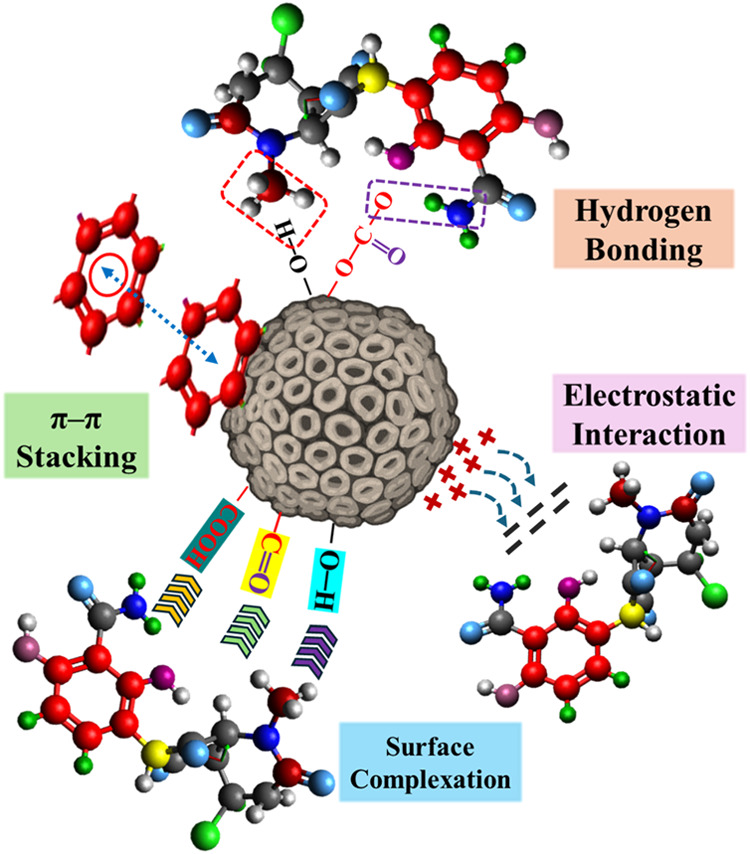
Adsorption mechanisms of TC onto carbonaceous adsorbents (TC structure created using Avogadro 1.2.0; other elements designed in BioRender).

### 7.1 π–π Interaction

The dominant mechanism of TC adsorption depends on the properties of the adsorbent and on conditions such as pH and ionic strength. For many carbon-based materials, π–π interactions, especially π–π electron donor–acceptor (EDA) interactions, play a key role. TC has conjugated aromatic rings that can stack face-to-face or edge-to-face with π-rich carbon surfaces. These interactions are stronger when the carbon is more graphitized, carries surface groups such as −OH and −COOH, and the pH is near neutral, which helps maintain the planar structure of TC. In doped carbons, partial charge transfer in π–π EDA interactions further increases adsorption. Ding et al. studied TC adsorption on carbon materials and emphasized the importance of π–π EDA interactions, especially in 2D carbon adsorbents. Polarized graphite provides electron-rich regions as active sites, while the carbonyl-rich conjugated ketene structure of TC behaves as an electron acceptor. This pairing promotes strong π–π EDA interactions and leads to high TC uptake.^89^

### 7.2 Hydrogen Bonding

Hydrogen bonding is one of the main pathways for TC adsorption. It occurs between the polar functional groups of TC (−OH, −NH_2_, −C=O) and oxygen-containing groups (−OH, −COOH) on the adsorbent surface. These groups can act as hydrogen bond donors or acceptors, helping TC molecules attach and stay on the surface, which increases adsorption capacity. The strength of hydrogen bonding depends on factors such as SSA, pore structure, and the amount of surface functional groups, and it is usually favored at neutral to slightly alkaline pH.^90^ Hydrogen bonding often works together with π–π stacking and electrostatic interactions to improve TC removal. For example, Li et al. studied TC adsorption on lignin-derived hierarchical porous carbon (LPHC) and showed that the abundant oxygen-containing surface groups (C=O, C–O, −OH) on LPHC formed hydrogen bonds with the −OH and −C=O functionalities of TC. Spectroscopic evidence (changes in the intensity and position of O–H/N–H and C=O/C–O bands) indicated that these hydrogen-bonding interactions were a major contributor to TC uptake.^91^

### 7.3 Electrostatic Interactions

Electrostatic interactions are an important factor in TC adsorption because TC is amphoteric and can exist in cationic, zwitterionic, or anionic forms depending on pH. At low pH (≤3), both TC and many adsorbents are positively charged, so electrostatic repulsion reduces adsorption. Between pH 3–6, TC is mainly zwitterionic, which can favor adsorption on weakly charged surfaces. At higher pH (≥8), TC is mainly anionic and can be strongly attracted to positively charged adsorbents. He et al. showed that electrostatic attraction near pH 6 greatly enhanced TC adsorption, whereas repulsion at acidic and highly alkaline pH reduced uptake.^92^ Liu et al. also found that, under alkaline conditions, electrostatic interactions were the main contributor to TC adsorption on grape leaf-based BC prepared at 900 °C, with changes in surface charge and TC speciation strongly controlling removal performance.^93^

### 7.4 Surface Complexation

Carbon materials contain many oxygen-containing functional groups (−OH, −COOH, −C=O) that can interact with the polar groups of TC (−OH, −NH_2_, >C=O) through ligand exchange or coordination, especially at neutral to slightly acidic pH, where TC is mainly in its zwitterionic form. Under these conditions, inner-sphere complexes can form by direct bonding between TC and surface groups, which contributes strongly to chemisorption and improves adsorption capacity, selectivity, and stability, while outer-sphere complexes based on electrostatic attraction play a smaller role.^94^ As shown in Figure 4, this pH dependence helps explain the generally better performance near neutral pH. In line with this, Ngoc et al. reported that hydrochar-derived ACs prepared from glucose solution by hydrothermal treatment and from ZnCl_2_-activated teak sawdust hydrochar showed enhanced TC adsorption due to their higher content of oxygenated surface groups, which favored inner-sphere complex formation and efficient TC uptake near neutral pH.^95^

## 8 Recent Advances in TC Removal

Recent advances in TC removal have focused on tailoring the physicochemical properties of carbon-based adsorbents to boost their adsorption efficiency. Key strategies include surface chemistry modifications to introduce active functional groups, pore size engineering to optimize accessibility and diffusion of TC molecules, and the development of multifunctional composites that enhance structural properties and reusability. These approaches have collectively contributed to significant improvements in adsorption performance and offer valuable insights for designing next-generation adsorbents. The following sections elaborate on each of these strategies in detail.

### 8.1 Surface Chemistry Modifications

Surface modification of carbon-based adsorbents by oxidation, amination, or metal oxide loading can improve TC adsorption by introducing functional groups such as −OH, −COOH, −NH_2_, and PO_4_^3–^. These groups increase active site density, tune surface charge, and strengthen hydrogen bonding, π–π stacking, and electrostatic interactions in water.^5^ For example, Quyen et al. prepared a magnetic AC/Fe_3_O_4_ composite from rice husk ash. The AC was first treated to remove silica and then acid-activated, which enriched the surface with oxygen-containing groups, especially −COO^–^. These groups then coordinated with Fe^2+^ and Fe^3+^ during coprecipitation, leading to uniform Fe_3_O_4_ deposition. The modified surface promoted electrostatic and complexation interactions with TC, and the Fe_3_O_4_ phase allowed easy magnetic separation of the adsorbent from solution.^102^ In a related BC system, Wu et al. synthesized ammonium persulfate (APS)-activated chitosan biochar (APS@CHI) using ammonium persulfate as an oxidizing agent. APS treatment introduced additional oxygen-containing groups (C=O, −COOH) and nitrogen functionalities (pyrrolic N-5 and pyridinic N-6) through oxidation and heteroatom doping. These changes increased surface reactivity and electron-donating capacity, improved TC binding (as illustrated in Figure 5), and, together with the high SSA and functional group density, made the material a strong candidate for antibiotic removal.^103^

**Figure 5:**
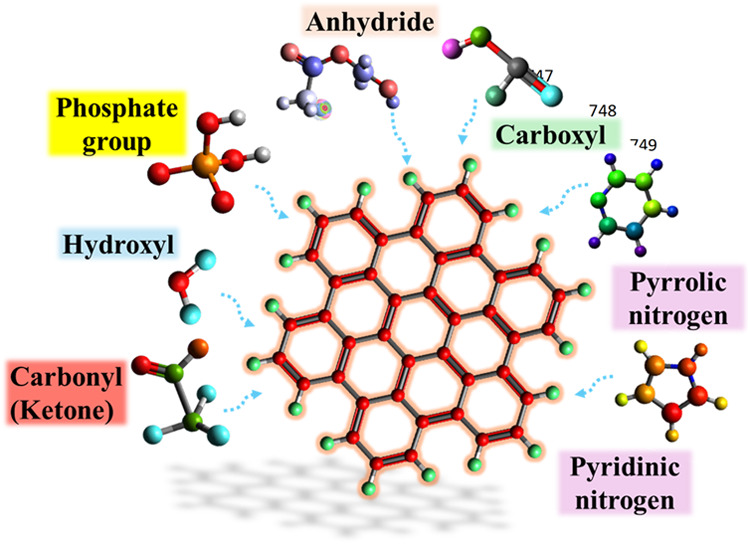
Schematic representation of surface functionalization in BC and AC for enhanced adsorption (Created using Avogadro 1.2.0).

### 8.2 Pore Size Modification

The pore size distribution of carbon-based materials strongly affects TC adsorption because TC has relatively large molecular dimensions. To improve access and diffusion of TC molecules, many recent studies have tuned the pore structure either during synthesis or by post-treatment. For AC, Wang et al. prepared 3D porous carbon from glucose and ammonium chloride by pyrolysis at 900 °C under argon, followed by controlled oxidation in air at 350, 400, and 450 °C. The resulting carbons had mainly micropores with an average pore size of about 2.5 nm, which is suitable for TC adsorption.^107^

Similar pore engineering in BC has also been effective. Du et al. used keratin-rich bovine horn as a precursor, first thermally pretreated it at 160–200 °C and then activated it with KOH at 900 °C. The pretreatment temperature strongly affected the final structure and porosity. The sample pretreated at 180 °C showed the best properties, with an ultrahigh SSA (2806 m^2^/g), a larger average pore diameter (2.68 nm), and a well-developed mesoporous structure. These features were linked to partial oxidation of disulfide bonds to sulfonic/sulfone groups and an increase in β-sheet content, which helped maintain structural integrity during activation.^108^

### 8.3 Composite Materials for Enhanced TC Adsorption

Recent work on TC removal has focused on engineered carbon materials combined with functional additives. For example, Fe_2_O_3_-loaded carbon prepared from rubber fig leaves showed higher TC adsorption because of the combined effect of its large SSA and magnetic properties.^109^ A multifunctional composite made by combining AC from *Camellia oleifera* shells with a magnetic MnO_2_/MCM-41@Fe_3_O_4_ phase, encapsulated in alginate and calcium chloride, also showed improved TC removal, mainly due to its mesoporous structure, high SSA, and easy magnetic separation.^110^ Similar progress has been made with BC-based composites. A BC-ceramsite material produced from pine sawdust, fly ash, red mud, and MgO by staged anoxic sintering achieved good TC removal through its porous structure and mineral-assisted adsorption.^111^ In another study, nitrogen-doped porous BC derived from ZIF-67 grown on polydopamine-coated loofah exhibited high TC uptake, attributed to its hierarchical pores and cobalt-based active sites.^112^

## 9 Future Scope and Perspectives

The continuous presence of TC and other antibiotics in water bodies is now recognized as a serious risk for both the environment and public health. Among the different treatment options, carbon-based materials such as AC and BC have attracted a lot of attention as adsorbents. They are mainly preferred because of their high specific surface area, adjustable pore structure, and rich surface functional groups, which together make them suitable for the selective removal of TC. Despite these advantages, some issues still limit their large-scale use. A major problem is the complex and often inconsistent synthesis of AC and BC. It is difficult to obtain materials with uniform morphology, stable surface chemistry, and reproducible properties from batch to batch, particularly when chemical modification or composite formation is involved. Therefore, future work should focus on greener, scalable, and low-cost preparation routes based on optimized pyrolysis and hydrothermal carbonization. In particular, efforts should prioritize scaling these processes in continuous or semicontinuous reactors and improving energy integration. Coupling production with locally available biomass residues can help to obtain carbon materials with more uniform properties at lower cost and with a reduced overall environmental burden. Another important research need is to evaluate the long-term behavior and reusability of these materials under more realistic conditions. Most available studies are carried out in controlled laboratory systems and do not fully reflect variations in pH, the presence of coexisting ions, natural organic matter, or microbial activity in real wastewater. More detailed investigations on the stability of AC and BC over repeated adsorption–desorption cycles, as well as on the possible leaching of incorporated metal species, are required to confirm their environmental safety and sustained adsorption performance.

In addition to plain carbon materials, hybrid adsorbents based on AC or BC combined with metal oxides or doped with heteroatoms are also being explored. These modified carbons often show higher removal efficiency because of combined effects, such as stronger adsorption affinity and higher surface reactivity. Such multifunctional materials may be useful in future treatment systems dealing with complex mixtures of pollutants. To move from lab studies to practical use, future work should include techno-economic analysis, life-cycle assessment, and pilot-scale trials in different types of wastewaters. In the long run, developing AC- and BC-based adsorbents that are selective, easy to regenerate, and environmentally safe will be important for the sustainable removal of TC from contaminated water.

## 10 Conclusion

This review shows that biomass-based AC and BC can play an important role in removing TC from water. They are relatively low-cost, can be tuned through surface modification, and are often produced by routes that are more environmentally friendly than many synthetic adsorbents. The removal of TC mainly depends on interactions such as π–π stacking, hydrogen bonding, and surface complexation. The way AC and BC are synthesized and activated strongly affects their surface area, pore structure, and functional groups, and therefore their adsorption behavior. In addition, solution conditions such as pH, ionic strength, and temperature change both the surface properties of the adsorbent and the speciation of TC, which together control the overall uptake. Surface functionalization, for example, by chemical doping or introducing heteroatoms, has been shown to improve selectivity and make regeneration more feasible. Although many lab-scale studies report promising results, several gaps remain, especially in tests with real wastewater, regeneration over many cycles, and long-term stability. Future work should focus on pilot-scale studies, life-cycle assessment, and ways to integrate these materials into existing treatment units so that their potential in advanced wastewater treatment can be fully demonstrated.

## Data Availability

Relevant data will be made available upon request.

## References

[1] Xu H., Wang S., Chen M., Xu X., Li B. (2026). Scalable bamboo-waste
solar evaporators for low-cost and sustainable desalination. Compos. Part B Eng..

[2] Ren S., Wang S., Liu Y., Wang Y., Gao F., Dai Y. (2023). A review on current
pollution and removal methods of tetracycline
in soil. Sep. Sci. Technol..

[3] Priya S. S., Radha K. V. (2017). A review on the
adsorption studies of tetracycline
onto various types of adsorbents. Chem. Eng.
Commun..

[4] Wang Y., Wu G. (2023). Leveraging anaerobic biodegradation
of tetracycline in anaerobic
digestion systems with different operational modes. Environ. Technol. Innovation.

[5] Yan L., Song X., Miao J., Ma Y., Zhao T., Yin M. (2024). Removal of tetracycline from water
by adsorption with biochar: A
review. J. Water Process Eng..

[6] Zhou X., Liu B., Chen Y., Guo L., Wei G. (2020). Carbon nanofiber-based
three-dimensional nanomaterials for energy and environmental applications. Mater. Adv..

[7] Houghton E. E., Yapi L., Haneklaus N., Brink H. G., Tichapondwa S. M. (2025). Coal Fly
Ash-Based Adsorbents for Tetracycline Removal: Comparative Insights
into Modification and Zeolite Conversion. J.
Xenobiot..

[8] Patel M., Kumar R., Kishor K., Mlsna T., Pittman C. U., Mohan D. (2019). Pharmaceuticals of emerging concern
in aquatic systems: chemistry, occurrence, effects, and removal methods. Chem. Rev..

[9] Daghrir R., Drogui P. (2013). Tetracycline antibiotics
in the environment: a review. Environ. Chem.
Lett..

[10] Gopal G., KVG R., Ravikumar K. V. G., M S., Salma M., J L. A. A., Chandrasekaran N., Chandrasekaran N., Mukherjee A. (2020). Green synthesized Fe/Pd and in-situ Bentonite-Fe/Pd
composite for efficient tetracycline removal. J. Environ. Chem. Eng..

[11] Sun W., Qian X., Gu J., Wang X.-J., Duan M.-L. (2016). Mechanism
and effect of temperature on variations in antibiotic resistance genes
during anaerobic digestion of dairy manure. Sci. Rep..

[12] Javid A., Mesdaghinia A., Nasseri S., Mahvi A. H., Alimohammadi M., Gharibi H. (2016). Assessment of tetracycline contamination in surface
and groundwater resources proximal to animal farming houses in Tehran,
Iran. J. Environ. Health Sci. Eng..

[13] Karthikeyan K. G., Meyer M. T. (2006). Occurrence of antibiotics
in wastewater treatment facilities
in Wisconsin, USA. Sci. Total Environ..

[14] Burke V., Richter D., Greskowiak J., Mehrtens A., Schulz L., Massmann G. (2016). Occurrence of antibiotics in surface and groundwater
of a drinking water catchment area in Germany. Water Environ. Res..

[15] Tran N. H., Hoang L., Nghiem L. D. (2019). Occurrence and risk
assessment of multiple classes of antibiotics in urban canals and
lakes in Hanoi, Vietnam. Sci. Total Environ..

[16] Wang Z., Chen Q., Zhang J. (2019). Characterization and
source identification of tetracycline antibiotics in the drinking
water sources of the lower Yangtze River. J.
Environ. Manage..

[17] Thomas K. V., Dye C., Schlabach M., Langford K. H. (2007). Source to sink tracking of selected
human pharmaceuticals from two Oslo city hospitals and a wastewater
treatment works. J. Environ. Monit..

[18] Hanna N., Tamhankar A. J., Lundborg C. S. (2023). Antibiotic concentrations and antibiotic
resistance in aquatic environments of the WHO Western Pacific and
South-East Asia regions: a systematic review and probabilistic environmental
hazard assessment. Lancet Planet. Health.

[19] Pena A., Paulo M., Silva L. J. G., Seifrtová M., Lino C. M., Solich P. (2010). Tetracycline antibiotics in hospital
and municipal wastewaters: a pilot study in Portugal. Anal. Bioanal. Chem..

[20] Yan C., Yang Y., Zhou J. (2013). Antibiotics in the surface
water of the Yangtze Estuary: occurrence, distribution and risk assessment. Environ. Pollut..

[21] Antos J., Piosik M., Ginter-Kramarczyk D., Zembrzuska J., Kruszelnicka I. (2024). Tetracyclines contamination in European
aquatic environments:
A comprehensive review of occurrence, fate, and removal techniques. Chemosphere.

[22] Zhou H., Lu Z., Liu X. (2024). Environmentally Relevant Concentrations of
Tetracycline Promote Horizontal Transfer of Antimicrobial Resistance
Genes via Plasmid-Mediated Conjugation. Foods.

[23] Taşkan E. (2016). Effect of
tetracycline antibiotics on performance and microbial community of
algal photo-bioreactor. Appl. Biochem. Biotechnol..

[24] Benhadj M., Alouiz I., Amarouch M. Y., Sennoune M., Mazouzi D. (2026). From lignocellulosic
biomass to activated carbon: Impact of composition, structure and
activation on the adsorption of organic pollutants. Sci. Afr..

[25] Hu Z., Srinivasan M. P., Ni Y. (2001). Novel activation process
for preparing
highly microporous and mesoporous activated carbons. Carbon.

[26] Opoku B. K., Isaac A., Micheal A. A., Bentum J. K., Muyoma W. P. (2021). Characterization
of chemically activated carbons produced from coconut and palm kernel
shells using SEM and FTIR analyses. Am. J. Appl.
Chem..

[27] Danish M., Pin Z., Ziyang L. (2022). Preparation and characterization of banana
trunk activated carbon using H3PO4 activation: A rotatable central
composite design approach. Mater. Chem. Phys..

[28] Wang S., Lee Y. R., Won Y. (2022). Development of high-performance
adsorbent using KOH-impregnated rice husk-based activated carbon for
indoor CO2 adsorption. Chem. Eng. J..

[29] Wazir A. H., Wazir I. U., Wazir A. M. (2024). Preparation
and characterization
of rice husk based physical activated carbon. Energy Sources, Part A.

[30] Yang Q., Wu P., Liu J. (2020). Batch
interaction of emerging tetracycline
contaminant with novel phosphoric acid activated corn straw porous
carbon: Adsorption rate and nature of mechanism. Environ. Res..

[31] Wang J., Lei S., Liang L. (2020). Preparation of porous
activated carbon from semi-coke
by high temperature activation with KOH for the high-efficiency adsorption
of aqueous tetracycline. Appl. Surf. Sci..

[32] Islam M. A., Nazal M. K., Akinpelu A. A. (2024). Novel activated carbon
derived from a sustainable and low-cost palm leaves biomass waste
for tetracycline removal: Adsorbent preparation, adsorption mechanisms
and real application. Diamond Relat. Mater..

[33] Zadeh Z. E., Abdulkhani A., Aboelazayem O., Saha B. (2020). Recent insights into
lignocellulosic biomass pyrolysis: A critical review on pretreatment,
characterization, and products upgrading. Processes.

[34] Zhang K., Zhang K., Li Y. (2023). Easily Pyrolyzable Biomass
Components Significantly Affect the Physicochemical Properties and
Water-Holding Capacity of the Pyrolyzed Biochar. Agriculture.

[35] Hu X., Zhang R., Xia B. (2022). Effect of Pyrolysis
Temperature on Removal Efficiency and Mechanisms of Hg­(II), Cd­(II),
and Pb (II) by Maize Straw Biochar. Sustainability.

[36] Uchimiya M., Hiradate S., Antal M. J. (2015). Dissolved phosphorus
speciation of flash carbonization, slow pyrolysis, and fast pyrolysis
biochars. ACS Sustainable Chem. Eng..

[37] Xiang W., Wan Y., Zhang X. (2020). Adsorption of tetracycline hydrochloride onto
ball-milled biochar: Governing factors and mechanisms. Chemosphere.

[38] Shao Z., Shuangbao, Wu S., Gao Y., Liu X., Dai Y. (2024). Two-step pyrolytic preparation of
biochar for the adsorption study of tetracycline in water. Environ. Res..

[39] Miao S., Sheng Y., Xia X., Chen W., Zhang H., Li K. (2025). A promising peanut shell-derived
biochar via ultrasonic-assisted
P/O codoping for simultaneous adsorption of cadmium­(II) and tetracycline
from aqueous solutions. J. Environ. Chem. Eng..

[40] Qin Y., Chai B., Wang C., Yan J., Fan G., Song G. (2022). Removal of
tetracycline onto KOH-activated biochar derived from rape
straw: Affecting factors, mechanisms and reusability inspection. Colloids Surf., A.

[41] Zhang Z., Jiang C., Li D. (2020). Micro-mesoporous activated
carbon simultaneously possessing large surface area and ultra-high
pore volume for efficiently adsorbing various VOCs. Carbon.

[42] Feng D., Guo D., Zhang Y. (2021). Functionalized
construction of biochar with
hierarchical pore structures and surface O-/N-containing groups for
phenol adsorption. Chem. Eng. J..

[43] Demiral İ., Samdan C., Demiral H. (2021). Enrichment
of the surface functional
groups of activated carbon by modification method. Surf. Interfaces.

[44] Jawad A. H., Saber S. E. M., Abdulhameed A. S., Reghioua A., ALOthman Z. A., Wilson L. D. (2022). Mesoporous activated
carbon from mangosteen (Garcinia
mangostana) peels by H3PO4 assisted microwave: Optimization, characterization,
and adsorption mechanism for methylene blue dye removal. Diamond Relat. Mater..

[45] Liu H., Xu G., Li G. (2020). The characteristics of pharmaceutical
sludge-derived
biochar and its application for the adsorption of tetracycline. Sci. Total Environ..

[46] Saghir S., Pu C., Fu E., Wang Y., Xiao Z. (2022). Synthesis of high surface
area porous biochar obtained from pistachio shells for the efficient
adsorption of organic dyes from polluted water. Surf. Interfaces.

[47] Long J., He P., Przystupa K., Wang Y., Kochan O. (2024). Preparation of Oily
Sludge-Derived Activated Carbon and Its Adsorption Performance for
Tetracycline Hydrochloride. Molecules.

[48] Liu J., Lin Q., Gao J., Jia X., Cai M., Liang Q. (2023). Adsorption
properties and mechanisms of methylene blue and tetracycline by nano-silica
biochar composites activated by KOH. Chemosphere.

[49] Zhang Y., Xu J., Xie Z. (2023). Enhanced adsorption performance of tetracycline
in aqueous solutions by KOH-modified peanut shell-derived biochar. Biomass Convers. Biorefin..

[50] Shi Q., Wang W., Zhang H. (2023). Porous biochar derived
from walnut shell as an efficient adsorbent for tetracycline removal. Bioresour. Technol..

[51] Jiang C., Zhang S., Wang L. (2025). Tetracycline adsorption
on nitrogen-doped furfural residue biochar: Kinetics, thermodynamics
and mechanism analysis. Colloids Surf., A.

[52] Wang J., Liu Y., Qian D. (2026). Efficient removal of tetracycline and norfloxacin
by KOH-activated carbon derived from Forsythia Fructus residue. J. Water Process Eng..

[53] Cao R., Sun S., Wang K. (2025). Natural rock asphalt-derived
N/S self-doped
hierarchical porous carbon for efficient removal of tetracycline. Surf. Interfaces.

[54] Jin J., Feng T., Gao R. (2018). Ultrahigh selective
adsorption of zwitterionic PPCPs both in the absence and presence
of humic acid: Performance and mechanism. J.
Hazard. Mater..

[55] Qiu L., Li C., Zhang S. (2024). Importance of oxygen-containing
functionalities
and pore structures of biochar in catalyzing pyrolysis of homologous
poplar. Chin. J. Chem. Eng..

[56] Choudhry Q., Fan M., Khan Z. E. (2025). Distinct impact of oxidation reactions
on the evolution of pore structures during H3PO4 activation of aliphatic-rich
poplar and aromatic-rich lignin. Biomass Bioenergy.

[57] Zheng Y., Wang J., Li D. (2021). Insight into the KOH/KMnO4
activation mechanism of oxygen-enriched hierarchical porous biochar
derived from biomass waste by in-situ pyrolysis for methylene blue
enhanced adsorption. J. Anal. Appl. Pyrolysis.

[58] Bazkiaee H. K., Sharifian S., Asasian-Kolur N., Najafi H., Pirbazari A. E., Harasek M. (2024). Efficient removal of tizanidine and tetracycline from
water: A single and competitive sorption approach using carboxymethyl
cellulose granulated iron-pillared clay. Appl.
Surf. Sci. Adv..

[59] Nie T., Hao P., Zhao Z., Zhou W., Zhu L. (2019). Effect of oxidation-induced
aging on the adsorption and co-adsorption of tetracycline and Cu2+
onto biochar. Sci. Total Environ..

[60] Cai Y., Liu L., Tian H., Yang Z., Luo X. (2019). Adsorption and desorption
performance and mechanism of tetracycline hydrochloride by activated
carbon-based adsorbents derived from sugar cane bagasse activated
with ZnCl2. Molecules.

[61] Ninh P. T. T., Tuyen L. T. N., Dat N. D., Dat N. D., Nguyen M. L., Nguyen M. L., Dong N. T., Dong N. T., Chao H.-P., Chao H. P., Tran H. N. (2023). Two-stage
preparation of highly mesoporous
carbon for super-adsorption of paracetamol and tetracycline in water:
Important contribution of pore filling and π-π interaction. Environ. Res..

[62] Li B., Huang Y., Wang Z., Li J., Liu Z., Fan S. (2021). Enhanced adsorption capacity of tetracycline
on tea waste biochar
with KHCO3 activation from aqueous solution. Environ. Sci. Pollut. Res..

[63] Salah Z., Aloulou H., Bhattacharyya S., Algieri C., Amar R. Ben. (2025). Potential
elimination of diclofenac sodium (DCF) from aqueous solution by adsorption
using orange peel waste-based activated carbon. Euro-Mediterr. J. Environ. Integr..

[64] Borthakur P., Aryafard M., Zara Z. (2021). Computational
and experimental
assessment of pH and specific ions on the solute solvent interactions
of clay-biochar composites towards tetracycline adsorption: Implications
on wastewater treatment. J. Environ. Manage..

[65] Yi B., Guo F., Wei Q. (2025). Functionalized Biochar: Mechanisms of Oxygen-Containing
Functional Groups in Water Pollutant Remediation. ACS ES&T Water.

[66] Mane P. V., Rego R. M., Yap P. L., Losic D., Kurkuri M. D. (2024). Unveiling
cutting-edge advances in high surface area porous materials for the
efficient removal of toxic metal ions from water. Prog. Mater. Sci..

[67] Sayğılı H., Güzel F. (2016). Effective
removal of tetracycline from aqueous solution
using activated carbon prepared from tomato (Lycopersicon esculentum
Mill.) industrial processing waste. Ecotoxicol.
Environ. Saf..

[68] Zhang D., He Q., Hu X., Zhang K., Chen C., Xue Y. (2021). Enhanced adsorption
for the removal of tetracycline hydrochloride (TC) using ball-milled
biochar derived from crayfish shell. Colloids
Surf., A.

[69] Zhao H., Li J., Li S., Jiang Y., Du L. (2024). Evaluation and optimization
of six adsorbents for removal of tetracycline from swine wastewater:
Experiments and response surface analysis. J.
Environ. Manage..

[70] Jia Y., Ou Y., Khanal S. K., Sun L., Shu W., Lu H. (2024). Biochar-based
strategies for antibiotics removal: mechanisms, factors, and application. ACS ES&T Eng..

[71] Nayak A., Karkare V., Dasari H., Sundarabal N. (2026). Removal of
Amoxicillin and Tetracycline antibiotics using high surface area activated
carbon derived from petroleum residues. Environ.
Res. Commun..

[72] Ghorbanian A., Mahroudi A., Eslami H., Dolatabadi M. (2020). Evaluation
of Activated Carbon Prepared from Pistachio Shell as an Adsorbent
for the Removal of Tetracycline Antibiotics from Aqueous Solution:
Kinetic and Isotherm Study. Pist. Heal. J..

[73] Naghipour D., Hoseinzadeh L., Taghavi K., Jaafari J., Amouei A. (2023). Effective
removal of tetracycline from aqueous solution using biochar prepared
from pine bark: isotherms, kinetics and thermodynamic analyses. Int. J. Environ. Anal. Chem..

[74] Zhao C., Ma J., Li Z., Xia H., Liu H., Yang Y. (2020). Highly enhanced
adsorption performance of tetracycline antibiotics on KOH-activated
biochar derived from reed plants. RSC Adv..

[75] Wang Z., Wang Y., Yu K., Zhang M., Ding T., Xu L. (2024). Insights into the adsorption
behavior of tetracycline in various
shaped carbon nanopores: Interplay between mass transfer and adsorption. Microporous Mesoporous Mater..

[76] Jiang Y., Tan P., Liu X.-Q., Sun L.-B. (2022). Process-oriented smart adsorbents:
tailoring the properties dynamically as demanded by adsorption/desorption. Acc. Chem. Res..

[77] Liu S., Pan M., Feng Z. (2020). Ultra-high adsorption of tetracycline antibiotics
on garlic skin-derived porous biomass carbon with high surface area. New J. Chem..

[78] Saremi F., Miroliaei M. R., Nejad M. S., Sheibani H. (2020). Adsorption of tetracycline
antibiotic from aqueous solutions onto vitamin B6-upgraded biochar
derived from date palm leaves. J. Mol. Liq..

[79] Egbosiuba T. C., Abdulkareem A., Kovo A. (2020). Ultrasonic enhanced
adsorption of methylene blue onto the optimized surface area of activated
carbon: Adsorption isotherm, kinetics and thermodynamics. Chem. Eng. Res. Des..

[80] Marzbali M. H., Esmaieli M., Abolghasemi H., Marzbali M. H. (2016). Tetracycline adsorption
by H3PO4-activated carbon produced from apricot nut shells: A batch
study. Process Saf. Environ. Prot..

[81] Li, X. Biochar Production from Flocculent and Granular Sludge: Revealing the Roles of Inorganics Originated from Seawater; Hong Kong University of Science and Technology: Hong Kong, 2020.

[82] Huang Z., Fang X., Wang S., Zhou N., Fan S. (2023). Effects of
KMnO4 pre-and post-treatments on biochar properties and its adsorption
of tetracycline. J. Mol. Liq..

[83] Kim D.-G., Boldbaatar S., Ko S.-O. (2022). Enhanced Adsorption of Tetracycline
by Thermal Modification of Coconut Shell-Based Activated Carbon. Int. J. Environ. Res. Public Health.

[84] Ma X., Xin Y., Yan Q. (2020). Adsorption Characteristics of Tetracycline
onto Biochars as Affected by Solution Chemistry Conditions and Ball
Milling Treatment. Water, Air, Soil Pollut..

[85] Islam M. A., Nazal M. K., Akinpelu A. A., Sajid M., Alhussain N. A., Ilyas M. (2024). High performance adsorptive removal of emerging contaminant paracetamol
using a sustainable biobased mesoporous activated carbon prepared
from palm leaves waste. J. Anal. Appl. Pyrolysis.

[86] Al-Ghouti M. A., Da’ana D. A. (2020). Guidelines
for the use and interpretation of adsorption
isotherm models: A review. J. Hazard. Mater..

[87] Varadharajan V., Senthilkumar D. S., Senthilkumar K. (2022). Process modeling and
toxicological evaluation of adsorption of tetracycline onto the magnetized
cotton dust biochar. J. Water Process Eng..

[88] Lin L., Ning L., Chen S. (2025). Ultra-large adsorption
capacity for tetracycline by defatted Schizochytrium limacinum residue
biochar. Algal Res..

[89] Ding W., Zhou G., Wen S. (2022). Two-dimensional activated
carbon nanosheets for rapid removal of tetracycline via strong π-π
electron donor receptor interactions. Bioresour.
Technol..

[90] Tian M., Lennox M. J., O’Malley A. J. (2021). Effect of pore geometry
on ultra-densified hydrogen in microporous carbons. Carbon.

[91] Li X., Li Y., Li Y., Zhang M., Zhu J. (2026). Synthesis
of Lignin-Derived
Hierarchical Porous Carbon via Hydrothermal–Phosphoric Acid
Synergistic Activation for Enhanced Adsorption of Tetracycline. Molecules.

[92] He T., Yang W., Wang Y., Yang J., Mei J., Cui S. (2025). The efficient
adsorption of tetracycline by carbon materials derived
from cobalt-doped NH2-MIL-125­(Ti) with synergistic effect. J. Water Process Eng..

[93] Liu D., Cai Y., Yu X., Wang Q. (2025). Removal of tetracycline
with grape
leaves–based biochar: adsorption properties and mechanism. Biomass Convers. Biorefin..

[94] Qin Q., Wu X., Chen L., Jiang Z., Xu Y. (2018). Simultaneous
removal
of tetracycline and Cu (II) by adsorption and coadsorption using oxidized
activated carbon. RSC Adv..

[95] Ngoc D. M., Hieu N. C., Trung N. H. (2023). Tetracycline Removal
from Water by Adsorption on Hydrochar and Hydrochar-Derived Activated
Carbon: Performance, Mechanism, and Cost Calculation. Sustainability.

[96] Longchar I. T., Sharma S., Umdor R. S., Bora P., Sinha D. (2025). Utilization
of activated Thysanolaena maxima biomass for the high-performance
removal of tetracycline antibiotic from wastewater: experimental optimization
and DFT simulation. Biomass Convers. Biorefin..

[97] Toffoli
de Oliveira J., de L. R., Costa C., Agnol G. D., Féris L. A. (2025). Optimizing Tetracycline Removal through Batch and Continuous
Adsorption on Modified Activated Carbon: A Bayesian Perspective and
Advanced Modeling Analysis. Ind. Eng. Chem.
Res..

[98] Bian S., Cai Z., Xing W. (2025). Microporous carbon derived from waste plastics
for efficient adsorption of tetracycline: Adsorption mechanism and
application potentials. Environ. Res..

[99] He N., Hu D., Xie H. (2025). Sustainable production of sawdust-derived porous
carbon: Distinguished roles of phosphates and polyphosphates on pore
evolution and tetracycline hydrochloride purification. Sep. Purif. Technol..

[100] Fu H., Song X., Liu Y. (2025). Preparation
and characterization
of a novel activated carbon from wheat dust and its adsorption mechanism
for tetracycline hydrochloride. Particuology.

[101] Dai H., Liu Z., Xu L. (2025). Remediation of tetracycline
contaminatedsoiland water phases by straw-derived biochar: Adsorption
performance and bacterial community evolution. Ind. Crops Prod..

[102] Quyen N. D. V., Tuyen T. N., Khieu D. Q. (2025). The
Composite of Activated Carbon/Magnetic Iron Oxide: Synthesis, Isothermal,
and Kinetic Studies of Ciprofloxacin and Tetracycline Adsorption From
a Binary-Component Solution. J. Nanotechnol..

[103] Wu H., Lv H., Yu Y., Du Y., Du D. (2025). Ammonium persulfate-triggered
modified chitosan biochar for co-adsorption of Cr­(VI) and tetracycline
antibiotics: Behavior and mechanisms. Int. J.
Biol. Macromol..

[104] Jang H. M., Yoo S., Choi Y. K., Park S., Kan E. (2018). Adsorption isotherm, kinetic modeling
and mechanism of tetracycline
on Pinus taeda-derived activated biochar. Bioresour.
Technol..

[105] Jang H. M., Kan E. (2019). Engineered biochar
from agricultural
waste for removal of tetracycline in water. Bioresour. Technol..

[106] Huang B., Huang D., Zheng Q. (2023). Enhanced
adsorption capacity of tetracycline on porous graphitic biochar with
an ultra-large surface area. RSC Adv..

[107] Wang S., Hao M., Xiao D., Zhang T., Li H., Chen Z. (2023). Synthesis
of porous carbon nanomaterials and their
application in tetracycline removal from aqueous solutions. Chin. J. Chem. Eng..

[108] Du Z., Liu Z., Zhao W., Zaman F., Peng Q., Huang Y. (2025). Structural engineering on keratin
to tailor porous structure of biochar
for high-efficiency tetracycline removal. Int.
J. Biol. Macromol..

[109] Vinayagam R., Quadras M., Varadavenkatesan T. (2023). Magnetic activated carbon synthesized using rubber fig tree leaves
for adsorptive removal of tetracycline from aqueous solutions. Environ. Res..

[110] Zheng Z., Shi R., Zhang X., Ni Y., Zhang H. (2024). Preparation of Activated Carbon-Reinforced Composite Beads Based
on MnO2/MCM-41@ Fe3O4 and Calcium Alginate for Efficient Removal of
Tetracycline in Aqueous Solutions. Polymers.

[111] Zhang K., Zhang L., Dong X., Zhao Y., Li F., Cen Q. (2023). Efficient adsorption of tetracycline hydrochloride
on biochar-ceramsite composite: Optimization of response surface methodology
and investigation of adsorption mechanism. Mater.
Today Sustainable.

[112] Wu Q., Yu W., Wu Y. (2025). Magnetic
porous cobalt-embedded
nitrogen-doped biochar derived from natural loofah cellulose for efficient
adsorption of tetracycline from water. Colloids
Surf., A.

